# Programs and processes for advancing pediatric acute kidney support therapy in hospitalized and critically ill children: a report from the 26th Acute Disease Quality Initiative (ADQI) consensus conference

**DOI:** 10.1007/s00467-023-06186-4

**Published:** 2023-11-06

**Authors:** Tara M. Neumayr, Benan Bayrakci, Rahul Chanchlani, Akash Deep, Jolyn Morgan, Ayse Akcan Arikan, Rajit K. Basu, Stuart L. Goldstein, David J. Askenazi, The ADQI 26 workgroup, Rashid Alobaidi, Sean M. Bagshaw, Matthew Barhight, Erin Barreto, O. N. Bignall Ray, Erica Bjornstad, Patrick Brophy, Jennifer Charlton, Andrea L. Conroy, Prasad Devarajan, Kristin Dolan, Dana Fuhrman, Katja M. Gist, Stephen M. Gorga, Jason H. Greenberg, Denise Hasson, Emma Heydari, Arpana Iyengar, Jennifer Jetton, Catherine Krawczeski, Leslie Meigs, Shina Menon, Catherine Morgan, Theresa Mottes, Zaccaria Ricci, David T. Selewski, Danielle Soranno, Natalja Stanski, Michelle Starr, Scott M. Sutherland, Jordan Symons, Marcelo Tavares, Molly Vega, Michael Zappitelli, Claudio Ronco, Ravindra L. Mehta, John Kellum, Marlies Ostermann

**Affiliations:** 1 Department of Pediatrics, Divisions of Pediatric Critical Care Medicine and Pediatric Nephrology, Washington University School of Medicine, St. Louis, MO USA; 2 Department of Pediatric Intensive Care Medicine, The Center for Life Support Practice and Research, Hacettepe University, Ankara, Türkiye; 3 Department of Pediatrics, Division of Pediatric Nephrology, McMaster University, McMaster Children’s Hospital, Hamilton, ON Canada; 4 Department of Women and Children’s Health, School of Life Course Sciences, King’s College London, London, UK; 5 Pediatric Intensive Care Unit, King’s College Hospital NHS Foundation Trust, London, UK; 6 Center for Acute Care Nephrology, Cincinnati Children’s Hospital Medical Center, Cincinnati, OH USA; 7 Department of Pediatrics, Divisions of Critical Care Medicine and Nephrology, Baylor College of Medicine, Houston, TX USA; 8 Department of Pediatrics, Division of Critical Care Medicine, Northwestern University Feinberg School of Medicine, Ann & Robert Lurie Children’s Hospital of Chicago, Chicago, IL USA; 9 Department of Pediatrics, Division of Nephrology & Hypertension, Cincinnati Children’s Hospital Medical Center, Cincinnati, OH USA; 10 Department of Pediatrics, Division of Pediatric Nephrology, Pediatric and Infant Center for Acute Nephrology, Children’s of Alabama, University of Alabama at Birmingham, Birmingham, AL USA

**Keywords:** AKI, CRRT, Dialysis, CVVH, Children, Critical care

## Abstract

Pediatric acute kidney support therapy (paKST) programs aim to reliably provide safe, effective, and timely extracorporeal supportive care for acutely and critically ill pediatric patients with acute kidney injury (AKI), fluid and electrolyte derangements, and/or toxin accumulation with a goal of improving both hospital-based and lifelong outcomes. Little is known about optimal ways to configure paKST teams and programs, pediatric-specific aspects of delivering high-quality paKST, strategies for transitioning from acute continuous modes of paKST to facilitate rehabilitation, or providing effective short- and long-term follow-up. As part of the 26th Acute Disease Quality Initiative Conference, the first to focus on a pediatric population, we summarize here the current state of knowledge in paKST programs and technology, identify key knowledge gaps in the field, and propose a framework for current best practices and future research in paKST.

## Introduction

Pediatric acute kidney support therapy (paKST) has become an important part of the care of acutely and critically ill neonates, infants, and children. The goal of a paKST program is to reliably provide safe, effective, and timely extracorporeal supportive care that optimizes both hospital-based and lifelong outcomes for pediatric patients with acute kidney injury (AKI), inadequate clearance of toxins, and/or fluid and electrolyte derangements in the context of organ failures and critical illness. While there is a significant body of literature describing populations of pediatric patients who receive kidney support therapy (KST) and their outcomes [1–5], much less is known about optimal structures to configure paKST teams and programs, pediatric-specific aspects of delivering high-quality paKST, strategies for transitioning away from continuous modes to intermittent or chronic modes or to dialysis-independent rehabilitative states, or the best approach to providing intermediate- and long-term follow-up which will enable early detection of subacute and chronic sequelae after paKST that may affect health and well-being over the child’s lifespan. As a result, paKST varies greatly across pediatric hospitals both in the composition and functioning of paKST programs and in the technical aspects and processes of paKST delivery. In addition, utilizing paKST platforms for non-kidney-related diseases is an active area of investigation with the potential to improve outcomes in severe and complex disease processes refractory to standard interventions.

The Acute Disease Quality Initiative (ADQI, formerly known as Acute Dialysis Quality Initiative) methodology and the overall consensus recommendations of the 26th ADQI have been published previously [6, 7]. A detailed description of the ADQI methodology is also available at www.adqi.net. The 26th ADQI Conference convened international experts on pediatric AKI, including nephrologists, intensivists, pharmacists, dieticians, a patient representative, and an expert in pediatric social determinants of health. Each member of the subgroup on paKST engaged with pre-conference work developing a list of preliminary questions and objectives and performing a systematic literature review around these key questions. Key questions and recommendations were rigorously refined during the in-person 26th ADQI meeting using input from the entire group of conference participants as well as subgroup breakout sessions and were put into final form in post-conference subgroup meetings.

**Fig. 1:**
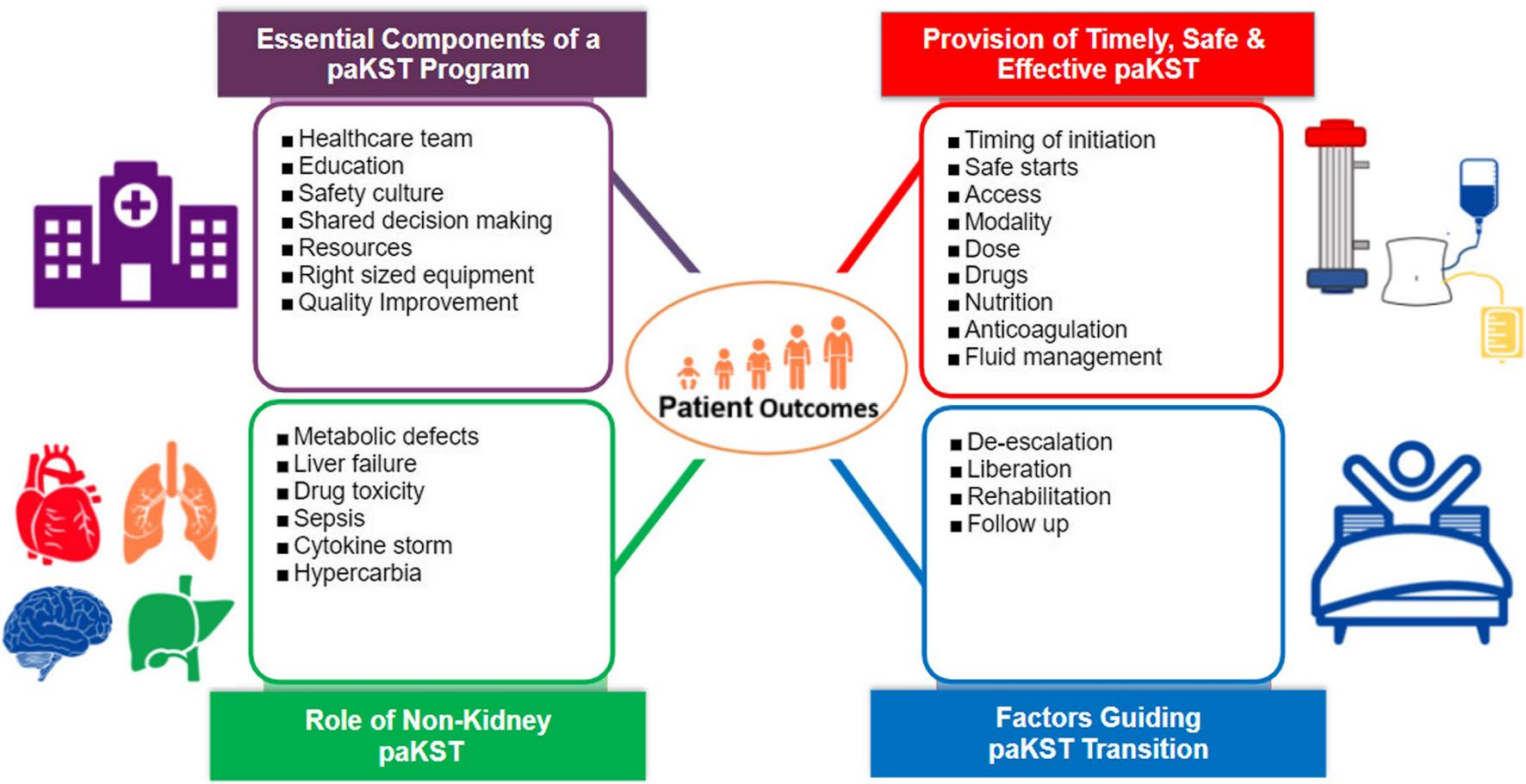
How paKST can be used to improve care and outcomes in children

Here, we summarize the current state of knowledge regarding paKST program structure, function, and technology, identify key knowledge gaps in this field, and propose a framework for current best practices and future inquiry in paKST. Our group identified more than 50 unresolved questions in paKST. We distilled those questions to ask: How can paKST be used to improve care and outcomes in children? We categorized our questions into four key areas (Fig. 1): (1) the essential components of a paKST program; (2) the provision of timely, safe, and effective paKST; (3) the factors guiding paKST de-escalation and liberation; (4) the role of paKST for non-kidney indications. We expand on the first three of these concepts below. The fourth was included in the 26th ADQI consensus report [7], but its more detailed treatment is beyond the scope of this report and will be addressed at an appropriate length separately.

## Building pediatric acute kidney support therapy programs

### Pediatric acute kidney support therapy program requirements

Well-functioning programs are fundamental to the reliable and consistent delivery of high-quality, complex procedures such as paKST. Specific components of a paKST program may vary by location and health care setting, and each program will need to determine the model that best suits its particular setting, patient population, and available KSTs. The model depends on institutional resources available (i.e., dialysis, nephrology, and intensive care unit (ICU)/critical care teams) and may be nephrology led, ICU led, or a hybrid of those.

The paKST program should be led by physician and nurse directors who can engage stakeholders and develop a multi-disciplinary team to.

- Create a culture of safety and transparency
- Secure resources of protected time, personnel, and equipment
- Obtain and maintain paKST equipment and supplies
- Track patient- and process-specific quality improvement (QI) data that will enable teams to understand and improve their processes and outcomes.

A list of suggested metrics is presented in Table 1, recognizing that this is neither an exhaustive list of potentially useful domains nor likely to be immutable over time. Individual programs may find some of them useful and others less so, and programs may find different metrics useful at different times. The principle that assessment practices should be matched to local needs is vital to a thoughtful and effective QI approach.

**Table 1:** Suggested quality improvement domains for clinical program performance assessment

Mortality	Infection rates
at 30 days	bacteremia
at 90 days	peritonitis
Dialysis-free kidney recovery	other
at ICU discharge	Vascular thrombosis rates
at hospital discharge	paKST filter life (continuous KST)
at 90 days	Fluid balance at paKST liberation/circuit discontinuation
ECMO duration	(as % of admission weight)
Length of mechanical ventilation	RBC transfusions during paKST (ml/kg)
Functional status	Platelet transfusions during paKST (ml/kg)
at hospital discharge	Outpatient follow up within 90 days
at 90 days	with Pediatric Nephrology
	with creatinine measurement
	with blood presssure measurement
	with urinalysis

The program’s medical and nursing directors should be well versed in bedside paKST care and have protected time dedicated to program development, education, and quality improvement. The paKST multidisciplinary leaders engage physicians, nurses, advance practice providers (including physician assistants and nurse practitioners), and the wider nephrology and critical care teams involved in the provision of paKST. Additional team members include pharmacists, nutritionists, social workers, surgeons and interventional radiologists, quality improvement/data specialists, and hospital administration personnel. The paKST team should meet regularly to provide updates; address potential safety events; budget for personnel and capital resources; develop education strategies; review and revise guidelines, policies, and procedures; and review center-specific quality data which will drive future projects and improvements.

Programs must choose where paKST care will be provided within the hospital, which may include dedicated hospital units or areas, the neonatal, pediatric, and cardiac intensive care units (ICU), and operating rooms. They must also choose what types of paKST they will provide—acute peritoneal dialysis (PD), acute intermittent hemodialysis (IHD), and/or acute continuous kidney support therapy modes—and whether they will provide paKST in tandem with other extracorporeal supportive therapies, including extracorporeal membrane oxygenation (ECMO), apheresis therapies, or extracorporeal liver support therapies. Updated and specific policies and procedures need to be in place for these services and must be easily accessible to all stakeholders.

In situations in which a hospital or team cannot provide safe and effective paKST, which may often occur with the smallest children and neonates, the program must develop communication channels, criteria, and processes to transfer care to centers (or units within their own center) that can provide such care [8].

An evidence-based approach to KST program-building is lacking. Key areas for investigation include development of resource- and needs-assessment tools, creation of communication aids within and among treatment teams, and establishment of educational approaches for on-boarding of new team members, maintenance of competency, and dissemination of new information or practice changes. Programs will need sufficient fluidity and responsiveness to adapt to changing circumstances and pressures, and as such will need an on-going QI framework to assess program performance and opportunities.

### Quality improvement initiatives in acute KST in neonates and children

Acute KSTs are highly technical and complex purification procedures that can be performed using the peritoneal membrane or through access to the blood. Services, equipment, and quality of care differ across centers. Unnecessary practice variations stemming from differences in operational models, knowledge gaps, and/or failure to adopt and implement best practices (i.e., how KST is prescribed, monitored, and delivered) lead to suboptimal outcomes [9, 10]. High-quality acute KST demands assurance systems to assess whether practices and interventions are occurring as intended to ensure optimal care delivery and outcomes. To date, lack of agreed-upon benchmarks to evaluate the processes of paKST delivery has hindered the establishment of a comprehensive quality control system [11, 12]. While the preponderance of KST research examines the application of therapy (i.e., patient selection, timing, modality, dose, and anticoagulation), there are limited data exploring the organizational structures or processes by which care is being delivered, leading to a lack of consensus for acceptable performance standards [11, 13]. Appropriate selection and integration of QI efforts into clinical practice will facilitate improvements in reliability and standardization of care. Efforts at reducing practice variability and developing new standards in pediatrics are emerging but have not yet been broadly accepted or adopted [11, 13–17].

As there are currently no available consensus benchmarks for optimal paKST, development and implementation of a quality framework should take high priority in research and QI efforts. As highlighted by the pediatric commentary to the 22nd ADQI [14], areas of particular interest include, but are not limited to.

- paKST program structures and formal training programs
- Treatment delivery benchmarks for circuit priming practices, delivered dose, fluid management, circuit life, and anticoagulation
- paKST-specific adverse events and outcomes, such as hemodynamic instability at circuit initiation, bleeding, transfusion requirements, catheter malfunction, and catheter-associated blood stream infections

QI guidelines for the neonatal and infant population may be of special importance in light of the specific challenges of paKST equipment and prescription in very small, physiologically immature critically ill patients. Assessments of hemodynamics at circuit initiation and long-term growth and development, especially in cases where paKST requirement is prolonged, are particularly important.

Validation of these QI standards can then be used to establish minimum acceptable performance criteria against which a paKST program can measure itself, driving further programmatic improvement [14].

### Optimizing pediatric acute kidney support therapy delivery

#### Timing of initiation in paKST

The basic questions: Is earlier KST in AKI better for patient outcomes? How early is early enough?—remain largely unanswered for the pediatric population. The field of critical care nephrology has been shaped by the recognition that the development of kidney injury and, in particular, the need for KST is associated with profound morbidity and mortality risk in critically ill patients. The potential impacts of the metabolic and fluid balance derangements resulting from AKI are rational targets for therapeutic intervention, leading to questions regarding the potential benefits of early KST for preventing, reducing, or correcting said metabolic and fluid abnormalities.

Evidence for timing of initiation in pediatrics is fairly sparse based on retrospective and/or observational data, but consistently demonstrates poorer outcomes in children with more severe kidney injury and/or fluid accumulation at the time of KST initiation in the ICU, on ECMO, or after cardiac surgery [18–20]. Inferences from recently conducted meta-analyses and multicenter randomized controlled trials in adults [21–28] are limited due to developmental physiologic differences and case mix. Focusing on AKI staging to define early/late KST [21, 24, 26, 27] introduces bias due to limitations of creatinine-based assessments of kidney function.

These uncertainties call for a personalized approach in deciding the optimal timing of initiation of KST for AKI to determine whether individuals with specific disease processes (such as cardiac surgery, sepsis, and acute respiratory distress syndrome) or clinical conditions (e.g., based on illness severity scores or organ dysfunction scores) would benefit from early KST.

Clinical thresholds of fluid balance/fluid overload, kidney injury biomarkers such as neutrophil gelatinase-associated lipocalin (NGAL) and TIMP-2*IGFBP-7, or a combination of clinical, functional, and/or biochemical markers (e.g., the renal angina index (RAI), the fluid overload kidney injury score (FOKIS), and the furosemide stress test (FST) [29–32]) may provide better tools for decisions on timing of initiation of KST and is currently an active area of research. Notably, in spite of decades of accumulated data demonstrating the correlation between fluid overload and poor outcomes, acknowledged in practice guidelines such as those for pediatric sepsis and ECMO management [33, 34], studies for which the primary indication for KST initiation is a threshold of fluid overload do not exist.

The challenges associated with developing a cohort of pediatric patients that is large enough to address any one of these questions in a randomized controlled trial are formidable, and novel approaches are needed. Moreover, clinically impactful outcomes besides mortality (e.g., duration of mechanical ventilation, ICU and hospital length of stay, early mobility, and global functional outcomes) are essential in future trials in order to drive widespread interest in and adoption of identified best practices.

#### Pediatric acute KST modality

Continuous KST modes are widely used in acute settings, but little is known about particular practice patterns within the field. A recent modified Delphi study queried paKST prescribers and found that continuous venovenous hemodiafiltration (CVVHDF) was the most commonly employed modality [35], but data on the associations of different modalities with patient outcomes are lacking. Whether continuous venovenous hemodialysis (CVVHD) or continuous venovenous hemofiltration (CVVH) are more beneficial than CVVHDF in certain disease states or patient populations or settings is unknown. Likewise, it is unknown whether there is a specific ratio of convective versus dialytic clearance that is most beneficial in acutely and critically ill pediatric patients or in specific clinical scenarios. Modes that combine some of the features of continuous KST and HD, such as prolonged intermittent kidney replacement therapy (PIKRT), may offer advantages in resource utilization and cost, coordination of testing and procedures, gradual liberation from KST, and/or patient rehabilitation efforts that have not yet been elucidated [36]. Similarly, well-established, less expensive modes such as PD may be preferable in some situations outside of post-cardiac surgery and resource-limited care, such as newly diagnosed kidney failure or other conditions in which a longer course of dialysis dependence is anticipated. The clinical and contextual factors that dictate choice of one modality over another are largely unknown and warrant further investigation.

#### Hemofilter selection in pediatric acute KST

In the treatment of AKI and fluid overload, the choice of hemofilter for continuous paKST has been guided by a few general principles: (1) the surface area of the filter should match as closely as possible to the patient’s body surface area; (2) the extracorporeal priming volume and blood flow rates for the filter set should be as minimal as possible; (3) the chosen filter should provide good clearance characteristics for small- and middle-sized particles; and (4) the membrane should be as biocompatible as possible, limiting the likelihood of bradykinin-release syndrome upon circuit initiation. The challenges in obtaining a filter that met all of these criteria, especially for our smallest and sickest patients, have led to a number of adaptations in clinical use, as well as on-going efforts to design and implement right-sized KST machines and filters specifically for that population. The current state of those efforts is represented in Table 2.

**Table 2:** Technical aspects of novel KST machines for neonates and children

	ECV (ml)	Filter (m^2^)	Q_b_ (ml/min)	UF (ml/h)	Hemodynamic Monitoring	Accuracy	Modality	Duration
Prismaflex							CVVH	
HF-20 (PAES)	60	0.2	10–100 (+ 2)	0–500 (+ 10)	NO	± 10% if UF setting	CVVHD	72 h (recommende)
M-60/ST60 (AN-59)	93	0.6	50–200 (+ 10)				CVVHDF	
Aquadex™	33	0.12	10–40 (+ 5)	0–500 (+ 10)	HCT and SV02	10%**	CVVH	
NIDUS	< 17	0.045	5 (single lumen)	0–60 (+ 1)	NO	< 0.25%	CVVHD	72 h
	26	0.075	2–50 (+ 1)	0–150	NO	± 30 g/day	CVVHD	72 h
	32	0.15		0–250			CVVH	24 h
	41	0.25		0–600			VCCHD	

*ECV* extracorporeal volume, *Filter (m*^*2*^*)* surface area of the filter, *UF* ultrafiltration, *HCT* hematocrit, *SVO2* mixed venous oxygen saturation, *CVVH* continuous venovenous hemofiltration, *CVVHD* continuous venovenous hemodialysis, *CVVHDF* continuous venovenous hemodiafiltration

^*^Experimental/not commercially available, **unpublished data suggest the margin of error to be < 3% at 350 ml/h

Outcomes in specific disease states, however, may be modifiable if the filter choices available offer the ability to match disease pathogenesis to clearance characteristics. This idea has been particularly tantalizing in patients with severe sepsis/septic shock and those with liver failure. Initial findings that the AN-69 filter may have the advantage of improved cytokine clearance in sepsis have been offset by the increased risk of bradykinin-release syndrome when this filter is used [37]. As a result, a number of filters have been designed and are currently being tested to achieve cytokine and/or endotoxin removal without inducing negative hemodynamic consequences; these include the oXiris membrane, the polymethylmethacrylate (PMMA) membrane, the CytoSorb cartridge, and the selective cytopheretic device. Similarly, charcoal and albumin-coated filters have been developed for liver failure patients in the hope of providing improved clearance of protein-bound solutes and toxins while awaiting clinical recovery or liver transplant. Tandem therapies combining paKST with apheresis potentially offer another pathway to improve outcomes with extracorporeal support in these patients. These issues will be the subject of an upcoming submission from our group, where they can be addressed in appropriate depth.

#### Pediatric acute KST dosing

Effluent volume remains the best method to assess dosing in paKST. The recommended dose of 2–3 L/1.73 m^2^/h of effluent was derived from expert opinion with little empiric basis when there was active debate in adult dosing between 25 and 45 ml/kg/h. Two large, randomized clinical trials demonstrated that higher doses are not superior to standard dosing [38], forming the basis for the current Kidney Disease Improving Global Outcomes (KDIGO) AKI guidelines establishing 20–25 ml/kg/h as the target delivered effluent dose in adults [39]. Similar studies are lacking in children. Analogous doses may lead to a higher intensity of treatment that compromises antimicrobial exposure and nutritional/mineral/vitamin adequacy. For example, in a 10-kg infant/child, an effluent rate of 2 L/1.73 m^2^/h corresponds to nearly 60 ml/kg/h, which is considered high-volume therapy in adults. High-volume therapy does not confer any clinical benefits in adults and may be associated with undesirable side effects such as more frequent or high-dose electrolyte replacement (e.g., phosphorus, calcium, potassium) and undetected but clinically important loss of micronutrients and lower antibiotic levels [40–42]. Effects of high-volume KST on other high-priority therapeutic agents, including steroids, immunosuppressive drugs, anticonvulsants, inotropes, sedatives, analgesics, and antivirals, are needed to inform both drug-dosing recommendations in KST and the risks associated with current paKST practices. The importance of addressing nutritional losses and their replacement in critically ill pediatric patients requiring KST cannot be overstated.

In addition, an understanding of KST dosing in tandem therapies such as ECMO and apheresis, and strategies to address medication doses during “down times”—both planned and inadvertent—impact the balance between prescribed and delivered dose. How this impacts safe and effective delivery of paKST is an area for ongoing investigation [43–46].

#### Circuit priming and initiation in paKST

paKST can be associated with significant clinical complications such as hypotension, hypothermia, bleeding, unplanned circuit loss, and thrombocytopenia [47]. Information on interventions that can be performed immediately before KST initiation to prevent undesirable events and avoid further compromise in an already fragile infant or child is sparse.

Many complications at KST initiation arise from the need for large extra-corporeal volumes (ECV) in relation to the patient’s total blood volume (TBV). Right-sized circuits with smaller filters and smaller ECV (Table 2) reduce the need for blood primes and limit hypotensive events around the time of initiation in small children [48–51]. Blood priming is generally recommended when the ECV is > 10–15% of the TBV in an effort to avoid acute hemodilution that may cause hemodynamic instability at circuit starts. No study has definitively established the appropriate threshold for blood prime in paKST. Blood primes increase the complexity of initiation and come with added risks, such as allosensitization and risks inherent to the use of stored blood which can cause hyperkalemia, hypocalcemia, and acidosis. The team must prepare for these issues proactively to prevent or minimize worsening hemodynamic instability. Various blood priming protocols exist to yield a more “physiologic” prime solution; safety, efficacy, and clinical consequences of these approaches need to be explored to determine which, if any, of them can be widely recommended and implemented [48, 49, 52–55]. In the meantime, the specific procedure for small children who may benefit from a blood prime initiation should be standardized for the individual program and not dictated by the rounding providers/team.

#### Vascular access for paKST

Well-functioning vascular access is a prerequisite for effective delivery of paKST, and temporary central venous catheters (CVCs) specifically designed for dialysis are the most utilized type of vascular access in children. Patient size and hemodynamic stability, underlying disease processes, expected type and duration of paKST, and center preferences and resources may all play a role in the choice and functional characteristics of vascular access. If paKST is likely to be needed for weeks, placement of a tunneled catheter may be preferable. In neonates requiring paKST, a tunneled double-lumen catheter that was cut to the desired length decreased complication rates compared to historic temporary catheters [56]. Two single-lumen catheters have been successfully used in small patients [57, 58].

Despite careful selection and placement, malfunctioning of vascular access—kinking/bending, leakage, thrombosis, infection, or high turbulence in inadequately sized catheters—contributes significantly to circuit failure necessitating a circuit/filter change [59]. Although pediatric recommendations for catheter size are available [60], optimal catheter sizing based on patient weight or height has not been established in pediatrics.

Catheter-related thrombosis/stenosis merits particular attention. Loss of vascular access—including permanent changes that preclude future use of that vein for permanent vascular or dialysis access—embolic events such as pulmonary embolism, and catheter-related bloodstream infections all have serious impact on the disease course and outcomes. Patients with liver failure, sepsis, and other pro-thrombotic conditions, as well as patients with longer duration of paKST treatment and particular catheter sizes and locations are at increased risk of thrombosis [61]. Evidence in non-dialysis ultrasound-guided CVC in adults suggests that a catheter-to-internal vessel size ratio > 45% increases thrombosis/stenosis risk [62]. Current KDIGO guidelines recommend ultrasound-guided catheter placement but do not specify an optimal catheter-to-vessel ratio [39]. Clinicians must carefully balance the benefits of good catheter flow against the risk of inducing thrombosis with an outsize catheter when choosing the size of vascular access for KST. Surface-modified catheters designed to lessen the risk of thrombosis [63] have not been widely adopted in clinical use. A recent pediatric study demonstrated a thrombosis rate of 7.4% with the preponderance in the smallest patients: 5 of the 6 patients with thrombosis were neonates [64]. We recommend future studies to establish guidelines for catheter sizing and length across the spectrum of pediatric patient sizes as well as for catheter-related thrombosis surveillance to allow for future development of more appropriate catheters for use in this high-risk population.

#### Circuit anticoagulation in paKST

Effective circuit anticoagulation remains one of the key determinants of successful administration of KST. The four most common continuous kidney replacement therapy anticoagulation strategies are systemic unfractionated heparin, low molecular weight heparin, prostacyclin, and regional citrate anticoagulation (RCA); nafamostat—a synthetic serine protease inhibitor—is emerging as a first-choice circuit anticoagulant in some centers, most notably in Asia [65]. Regional strategies that have minimal systemic effects have gained traction due to lower rates of bleeding events [66]. RCA has been incorporated into some KST machines in a semi-automated fashion with citrate infusion rates modulated with device software. When systemic anticoagulation is necessary, unfractionated heparin is most frequently used, but the lack of correlation between heparin dose and standard clinical monitoring tests such as activated partial thromboplastin time and activated clotting time poses a significant challenge for care teams. Alternative measures such as anti-Xa levels and thromboelastography have not demonstrated an advantage over traditional measures of heparin effect [67]. Elucidation of the best available anticoagulation and monitoring strategy or strategies, development of novel ones, fully automated integration of anticoagulation delivery into paKST machines, and creation of antithrombogenic membranes/circuit elements are all important areas for future study. Agents such as bivalirudin, argatroban, and nafamostat need to be systematically tested in children.

#### Fluid removal strategies in paKST

Treatment and/or prevention of clinically important fluid overload along with the ability to accommodate nutritional, medication, and blood product administration needs are frequent indications for paKST. Fluid balance needs may differ from patient to patient and from day to day or hour to hour in individual patients, necessitating a flexible and responsive approach to mechanical fluid removal. The assessment of appropriate rates of fluid removal in paKST patients is challenging, as the clinician must balance the risks and benefits of rapid fluid removal versus those of intravascular volume depletion. Incorporation of insensible fluid loss and the compartmentalization of excess volume between intravascular and extravascular spaces are important considerations in prescribing fluid removal rates.

Even in the presence of apparently stable hemodynamics, new regional cardiac stunning is common, often occurs within the first 4 h of therapy, and may be related to high ultrafiltration rates among adult critically ill KST patients [68]. The role of integrated ultrafiltration monitoring systems in acute KST is not clear and deserves further exploration; hematocrit sensors are considered standard in intermittent hemodialysis treatment [69, 70] but have not been studied in continuous KST. In critically ill patients, invasive hemodynamic monitoring and laboratory testing, such as venous oxygen saturation, may provide real-time guidance on fluid removal strategies in paKST and should be incorporated into future trials. Rigorous studies of the acceptable and safe range of ultrafiltration rates in paKST are needed to guide decision making; however, development of other decision support tools may be needed to account for the dynamic needs over the acute illness course for individual paKST patients.

Clear communication among paKST prescribers, critical care teams, and paKST operators is vital to treatment success. Shared approaches and language relating to evaluation of fluid balance, overarching clinical concerns and goals, daily and hourly fluid balance targets, and contingency planning should be developed by each paKST program. Clear communication with patients, families, and caregivers should also be emphasized within this framework. Daily (and sometimes more often) evaluations of the appropriate fluid removal rate are appropriate.

### The role of peritoneal dialysis in paKST

As the use of KST for AKI has increased among hospitalized children over the last two decades, the use of peritoneal dialysis (PD) has declined steadily within resource-abundant regions [71]. PD continues to have an important role in remote and/or resource-limited settings as well as in neonates after cardiac surgery. A 2013 meta-analysis did not demonstrate differences in outcomes between AKI patients treated with PD compared to blood-based KST modes [72]. PD offers many appealing aspects for paKST programs:

- Suitability in patients of nearly all ages, sizes, and disease states, including those with severe bleeding diatheses (in order to avoid the need for anticoagulation) or in whom obtaining vascular access is otherwise not feasible or safe
- Low cost—PD can be provided at three to five times less cost compared to blood-based acute KST [73]
- Ready transition from acute, inpatient therapy to chronic or rehabilitative therapy, including outpatient therapy

The International Society of Peritoneal Dialysis has developed detailed guidelines for the use of PD in both adult and pediatric AKI [74]. Existing barriers and challenges to PD use in various healthcare settings, optimal ways to dose acute PD for both clearance and fluid management—which may have significant differences from chronic PD dosing strategies—impact of PD on non-kidney disease, especially respiratory failure, and the roles of PD modalities such as tidal PD and continuous-flow PD are all rich areas for exploration in paKST care.

### Post-KST care for pediatric patients

#### Pediatric acute KST de-escalation and liberation

The question of when to stop or de-escalate KST may be second only to the question of when to start it in perplexity and importance. Available data demonstrate that, while kidney recovery may be incomplete, most children requiring dialysis for AKI will recover sufficiently to be dialysis independent [75, 76]. However, there is limited evidence to guide KST discontinuation. Current practices vary significantly and utilize assessment of hemodynamic stability, fluid balance status, urine output trends over time, and clinician estimates of the patient’s ability to maintain euvolemia and metabolic balance to make decisions about timing of discontinuation or transition to intermittent KST. A recent meta-analysis in adults identified 16 variables for predicting successful KST discontinuation; these variables can be categorized into physiologic parameters, such as hemodynamics and urine output, biochemical markers to evaluate glomerular filtration rate/kidney function, and novel kidney markers [77]. No comparable studies are available in children and given that there is no evidence to suggest that important differences between adults and children exist in this area, we recommend that future adult studies consider inclusion of pediatric patients where possible.

The consequences of too early or too late separation from KST among children with AKI are not yet defined. Clinically relevant outcomes such as end-of-therapy fluid balance, bloodstream infection rates, duration of mechanical ventilation, early mobility participation, sedative exposure, and ICU and hospital lengths of stay should be tracked and incorporated in paKST outcomes studies. As we increasingly appreciate the importance of early mobility and rehabilitation in critically ill patients [78], we will need to delineate ways in which KST needs and goals can harmonize with those initiatives. This may include earlier transition to intermittent modes of KST (3–4 h of HD or chronic PD) or designated prolonged (6–18 h) KST-free periods to facilitate rehabilitative therapies in appropriate patient populations. Markers that signal a high likelihood of success for discontinuation of KST—such as specific urine output thresholds (with or without diuretic challenge) or biochemical indicators—should be sought and implemented. Finally, for those patients who cannot readily liberate from KST, appropriate fluid and metabolic management strategies while kidney function remains impaired as well as strategies for transition to intermittent or chronic KST care—including both medical and administrative aspects of care—should be detailed.

#### Long-term monitoring and follow-up for paKST patients

Children who survive an episode of AKI requiring dialysis are at risk for adverse long-term outcomes such as chronic kidney disease, hypertension, and neurodevelopmental impairments, but only a minority receive pediatric nephrologist follow-up [75, 79–81]. It is unclear whether a subset of paKST patients at higher risk of these outcomes can be identified. Once known, the cadence with which these patients should receive follow-up evaluation and what tests those evaluations should contain should be delineated. Large longitudinal cohorts will be needed to answer these questions, allowing for development of clinical risk stratification tools to guide referrals and anticipatory guidance at the time of discharge from the index paKST event.

## Pediatric KST in resource-limited settings

The delivery of paKST in resource-limited and low-income settings has been mentioned in this report but deserves particular emphasis here. Innovative structures and processes will be required to deliver high-quality paKST when personnel, equipment, and physical space are limited and the processes of repair and resupply are slow and/or uncertain. Research efforts should focus on developing needs-assessment tools that can be flexibly applied to various circumstances, educational programs that promote effective troubleshooting and technical expertise along with quality assurance skills, identifying and using off-site and/or technology-based information resources, and paKST delivery that can be used in a broad swath of clinical scenarios while allowing both equipment and personnel to be deployed efficiently. As mentioned above, PD offers a number of advantages—wide applicability, safety, low cost, and transition from acute to chronic care—that may be particularly germane in resource-limited settings. In some settings, however, paKST teams may find that application of HD or PIKRT, which allow one piece of equipment to be used for more than one patient in the course of a day, better serves their needs. These teams will need data with which to guide the development of their particular programs and paKST delivery; they will also need funding for research to provide that data locally and/or regionally.

## Conclusion

Optimizing patient outcomes through provision of high-quality paKST requires programs and processes that are built on a robust evidence base, are right-sized for pediatric patients and their unique treatment specifications, are responsive to changing needs within individual patients as well as treatment environments, and are mindful of the broader context of acute illness and long-term care. Important work has been done in many of these areas to date, but fundamental questions remain. We have outlined key areas of paKST program building, clinical care, and follow-up that are ripe for exploration in both resource-abundant and resource-limited settings. Finding answers to these outstanding questions will drive the field forward and move us closer to better patient outcomes.
